# Semiautomated Workflow for Clinically Streamlined Glioma Parametric Response Mapping

**DOI:** 10.18383/j.tom.2016.00181

**Published:** 2016-12

**Authors:** Lauren Keith, Brian D. Ross, Craig J. Galbán, Gary D. Luker, Stefanie Galbán, Binsheng Zhao, Xiaotao Guo, Thomas L. Chenevert, Benjamin A. Hoff

**Affiliations:** 1 Imbio, LLC, Minneapolis, Minnesota;; 2 Department of Radiology, Center for Molecular Imaging, University of Michigan, Ann Arbor, Michigan; and; 3 Department of Radiology, Columbia University College of Physicians and Surgeons, New York, New York

**Keywords:** parametric response mapping, diffusion, glioma

## Abstract

Management of glioblastoma multiforme remains a challenging problem despite recent advances in targeted therapies. Timely assessment of therapeutic agents is hindered by the lack of standard quantitative imaging protocols for determining targeted response. Clinical response assessment for brain tumors is determined by volumetric changes assessed at 10 weeks post-treatment initiation. Further, current clinical criteria fail to use advanced quantitative imaging approaches, such as diffusion and perfusion magnetic resonance imaging. Development of the parametric response mapping (PRM) applied to diffusion-weighted magnetic resonance imaging has provided a sensitive and early biomarker of successful cytotoxic therapy in brain tumors while maintaining a spatial context within the tumor. Although PRM provides an earlier readout than volumetry and sometimes greater sensitivity compared with traditional whole-tumor diffusion statistics, it is not routinely used for patient management; an automated and standardized software for performing the analysis and for the generation of a clinical report document is required for this. We present a semiautomated and seamless workflow for image coregistration, segmentation, and PRM classification of glioblastoma multiforme diffusion-weighted magnetic resonance imaging scans. The software solution can be integrated using local hardware or performed remotely in the cloud while providing connectivity to existing picture archive and communication systems. This is an important step toward implementing PRM analysis of solid tumors in routine clinical practice.

## Introduction

Patients with glioblastoma multiforme (GBM), ∼15% of primary brain and central nervous system tumors and 46% of malignant brain and central nervous system tumors, face a dismal prognosis with a 1-year survival rate of 37%, dropping to 15% for a 2-year survival rate (1, 2). The current standard of treatment includes surgical resection followed by radiation therapy with concomitant administration of temozolomide. Despite the use of aggressive therapies, recurrence rate remains very high and patients undergo multiple rounds of chemotherapy and radiation therapy in an attempt to control local tumor growth (3). There remains significant interest in the development of therapies targeting unique oncogenic signaling pathways; therefore, robust methods for quantifying their efficacy are urgently required (4).

Magnetic resonance imaging (MRI) is the standard clinical method to monitor the extent of disease and the efficacy of treatment in patients with GBM. Determination of therapeutic response using the Macdonald criteria, the current standard of care, relies heavily on measurements of tumor dimensional changes 1 month following the conclusion of a complete treatment protocol, often 10–12 weeks after the commencement of therapy (5–7). This volumetric response is measured on serial contrast-enhanced T1-weighted (T1+C) magnetic resonance images, relying heavily on the radiologist's discernment of often-subtle characteristics of tumor margins. Defining tumor margins is complicated further by pseudoprogression, a phenomenon that occurs in up to two-thirds of patients treated with radiation therapy and concomitant administration of temozolomide within 3 months of completing therapy (7, 8). Treatment with these and other agents damages normal brain parenchyma, producing contrast enhancement and increased T2-weighted or fluid-attenuated inversion recovery (FLAIR) signal beyond the actual margins of the tumor. Current imaging sequences cannot reliably distinguish pseudoprogression from actual GBM progression. More recently, the Response Assessment in Neuro-Oncology Working Group proposed modifications in the response criteria for GBM to include stratification of nonenhancing lesions based on T2-weighted or FLAIR MRI (7). Although Response Assessment in Neuro-Oncology has advanced the assessment of glioma response to therapy, quantitative imaging approaches remain underutilized in the routine clinical setting.

Diffusion-weighted imaging (DWI) is an MRI technique that is sensitive to the microenvironmental mobility of water in tissues. Resulting apparent diffusion coefficients (ADCs) have been shown to inversely correlate with tumor cellular density in gliomas (9–12) and a variety of other tumor types (13–21). One of the earliest manifestations of a successful cytotoxic therapy is disruption of the cell membrane and overall integrity of tumor cells, resulting in decreased cellular density (22, 23) and thus a corresponding increase in ADCs. Because molecular and cellular changes precede gross tumor volume changes, DWI has the potential to provide an earlier biomarker of treatment response for management of patients with cancer, which has been confirmed in many studies (9, 12, 24–30). Longitudinal comparisons of tumor ADC have traditionally focused on mean tumor values at each time point. However, spatially varying intratumor heterogeneity of response has been observed as a major confounding factor in individual patient assessment (31–33). The continuing development of image-processing algorithms, such as the voxel-based parametric response mapping (PRM), offers improved opportunities for more sensitive characterization of disease and response (10, 34, 35).

PRM is an image analysis approach that uses spatially aligned, longitudinal images to classify response in a voxel-wise manner, resulting in improved sensitivity over volumetric measurements, particularly in the case of spatially dependent heterogeneous changes occurring in underlying imaging metrics during therapy. PRM of apparent diffusion coefficient maps (PRM_ADC_, previously known as the functional diffusion map) provides an early biomarker of treatment response in high-grade gliomas (34, 36) and maintains spatial context in the form of the classification map. PRM methods rely on accurate image segmentation and coregistration (31, 37), but the workflow remains time-intensive involving multiple custom algorithms. Experienced radiologists generally delineate regions of interest (ROIs) manually. However, this practice is both subjective and extremely time-consuming, particularly when performed on multiple slices spanning the tumor on high-resolution images to construct a volume of interest (VOI) (38). Routine implementation of PRM in clinical practice will require a user-friendly interface and automation to aid in significant clinical adoption.

Herein we have developed and evaluated a semiautomated PRM_ADC_ workflow and compared results with previously published data (12). The presented workflow was designed to enable practical implementation and use of PRM_ADC_ in the time-constrained clinical environment. We also highlight the potential for full automation of this image analysis method. Furthermore, this technology can be accessed through cloud-based servers, providing clinicians with a PRM summary statistical report to be used in clinical management but without the need for installing or maintaining local software and computing power. Overall, results present a software solution that provides a robust and semiautomated workflow, allowing for the evaluation of glioma therapy response using PRM_ADC_.

## Methods

### Patients and Therapy

Patient imaging data were acquired previously as a prospective study evaluating PRM_ADC_ as an imaging biomarker for glioma treatment response (12). 60 patients with high-grade gliomas undergoing chemo-radiotherapy were enrolled in the study.

Radiotherapy was delivered using 3-dimensional (3D) conformal therapy or intensity-modulated radiotherapy with at least 6-MV photons. Standard technique included a 2.0- to 2.5-cm margin on either the enhancing region on gadolinium-enhanced scans or the abnormal signal on T2-weighted scans to 46–50 Gy, with the gross tumor treated to a final median dose of 70 Gy in 6–7 weeks. 21 of these patients were treated on a phase 2 protocol of high-dose (60 Gy) radiation therapy with concurrent administration of temozolomide, dependent on clinical circumstances. Traditional radiologic response was assessed at week 10 by an experienced neuroradiologist using the Macdonald criteria.

### Imaging

Diffusion and standard anatomical MRI (FLAIR), T2-weighted, and gadolinium-enhanced T1-weighted (T1+C) images were acquired 1 week before and 1, 3, and 10 weeks after the commencement of radiation therapy with follow-up acquisitions every 2–3 months. Diffusion imaging was accomplished using a single-shot spin-echo echo-planar imaging protocol with diffusion weighting in 3 directions. MRI acquisitions were performed using 1 of the following 2 systems:

(1) 1.5 T MRI (n = 45; General Electric; Waukesha, Wisconsin).
(2) Achieva 3 T MRI (n = 15; Philips; Best, The Netherlands).

Acquisition parameters were as follows:

- 24 axial slices, 6-mm thick, 22 cm of field of view, 128^2^ matrix (voxel = 17.7 mm^3^), repetition time = 10 000 milliseconds, echo time = 71–100 milliseconds, and 1 average, with b values of 0 and 1000 s/mm^2^.
- 28 axial slices, 4-mm thick, 24 cm of field of view, 128^2^ matrix (voxel = 14 mm^3^), repetition time = 2636 milliseconds, echo time = 46 milliseconds, and b values of 0 (1 average) and 1000 s/mm^2^ (2 averages).

Parallel imaging (sensitivity encoding factor 3) was used at 3 T to reduce spatial distortion. The diffusion images for the 3 orthogonal directions were combined to calculate an ADC map.

ADC maps were calculated using the standard mono-exponential model: $ADC\;=\;\;\frac{\text{ln}(S_{1}/S_{2})}{b_{2}-b_{1}}$, where S_1_ is the image with b value b_1_ = 0 s/mm^2^ and S_2_ is the image with b value b_2_ = 1000 s/mm^2^. The diffusion-weighted images for the 3 orthogonal directions were combined to form S_2_.

### Semiautomated Tissue Segmentation

The semiautomated segmentation algorithm uses a combination of region-based active contours and level-set approaches (39, 40). The algorithm is initialized with a user-defined seed ROI, consisting of a rough outline of the tumor on a single slice, where the tumor has a relatively large area. Completely automated segmentation is then performed, extending the VOI to cover the full 3D tumor volume. Segmentations of contrast-enhancing tumor using T1+C images were used for analysis.

Seed ROIs were generated by a 2-dimensional binary dilation (3×) of a given slice from a radiologist-drawn VOI around the contrast-enhancing region of the tumor (compare “Manual” and “Seed” example images in Figure 3). For a given image, multiple seed ROIs were generated in this way to test the sensitivity of the segmentation algorithm to seed ROI identification. 737 seed ROIs were generated from 51 patients, each with 2 imaging time points (pretreatment and post-treatment).

**Figure 1.**
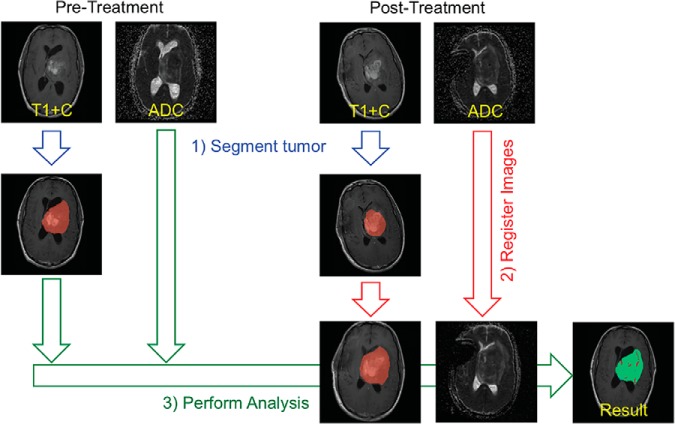
Software workflow is presented for processing and analysis of serial glioma diffusion data for parametric response mapping of apparent diffusion coefficients maps (PRM_ADC_). Images are first processed to generate an apparent diffusion coefficient (ADC) map and tumor segmentation. Then, follow-up images are spatially aligned with baseline images. Once ADC maps are aligned, they are further processed into a parametric response map, consisting of 3-voxel classifications: increased, decreased and unchanged ADC. Summary statistics for clinical evaluation consist of the tumor-relative volume of each classification as well as mean ADC at each time point.

**Figure 2.**
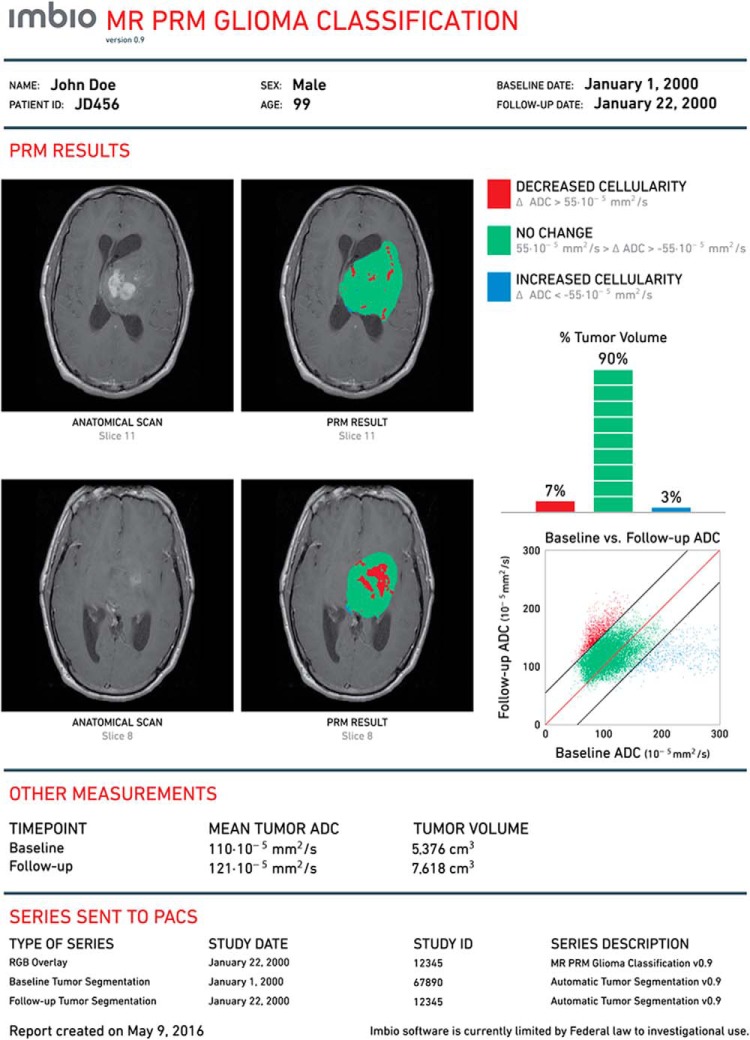
Example final report of parametric response mapping (PRM) results from the online software platform. This provides PRM-relative volumes as summary statistics along with key PRM overlays and scatterplot for insight into distribution patterns and spatial context. In addition, measures of mean tumor ADC and tumor volume are provided.

**Figure 3.**
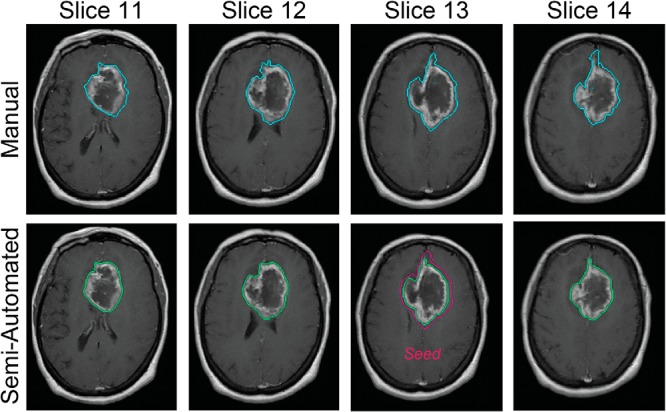
An example of automated segmentation reveals good agreement with the original manual segmentation. Manual segmentations drawn by an experienced radiologist are shown in the top row and semiautomated segmentation results in the second row. Generation of the semiautomated VOI began with the single-slice seed ROI shown in the bottom row (slice 13).

For each patient and time point, the seed ROI that yielded the optimal similarity was identified. Reproducibility of the segmentation was explored by applying a 1-voxel shift in each direction of this optimal seed ROI. For this analysis, shifted-seed segmentation volumes were compared with optimal seed segmentation volumes. Direct visual comparison of the segmentation volumes was also performed.

For PRM processing of 27 data sets, a 3× binary dilation on a single central slice of the provided VOI was used as a seed ROI for this algorithm, as mentioned above. For the remaining 15 data sets, seed ROIs were manually drawn on a central slice through the tumor.

### Automated Coregistration

An algorithm for nonlinear registration of serially acquired multimodal MRI images was developed which uses a 3-step process to register the baseline and follow-up anatomical images. The first step is a 3D block-matching algorithm with an intensity-based similarity metric that provides a global affine transformation. The second step uses a normalized mutual information metric and a B-spline interpolator to perform local registration of a down-sampled data set. Down-sampling is performed to increase the computational speed of the coarse, local registration. The control points for the B-spline interpolator are placed in a uniform grid with a spacing equal to 6 voxels. A bending energy penalty term is included to enforce a smooth solution. The final stage repeats the normalized mutual information and the B-spline local registration process with a full-resolution data set and a penalty term on the determinants of the Jacobian of the transformation are enforced to ensure a smooth transformation. The objective of this approach is to spatially align all serial imaging data to the pretreatment T1+C scan, referred to as the reference scan. In brief, serial T1+C images are spatially aligned, and the transformation matrix calculated is used to transform all post-ADC maps to the reference scan.

### Automated Parametric Response Mapping

The PRM analysis was performed following previously described methods. In brief, each voxel inside a VOI was categorized by the change in an ADC value (ΔADC) into 1 of 3 classifications: ΔADC > 55 × 10^−5^ mm^2^/s (red, “increased ADC”); ΔADC <−55 × 10^−5^ mm^2^/s (blue, “decreased ADC”); and −55 × 10^−5^ mm^2^/s < ΔADC < + 55 × 10^−5^ mm^2^/s (green, “unchanged ADC”). These threshold values were empirically determined as the 95% confidence intervals from normal contralateral brain tissues (34). The percentage fractional tumor volume for each of these categories is quantified and reported as a metric of response (PRM_ADC+_, PRM_ADC−_, and PRM_ADC0_, respectively).

Two separate tests were conducted to demonstrate the proposed workflow using previously published imaging data. First, segmentations on preregistered images were generated using semiautomated algorithms and compared against manual segmentations generated previously by experienced radiologists. This study used 49 coregistered patient data sets provided by the institution. Second, original imaging data were used directly from the institution's picture archive and communication system (PACS) and processed with the fully semiautomated workflow (segmentation, registration, and PRM). This study used 40 of the previously studied patients because of limitations querying all the required retrospective data from the PACS.

### Statistics

Segmentation comparisons were performed using the Dice coefficient of similarity (DCS), with values ranging from 0 (not at all similar) to 1 (exactly the same). Receiver operator curve (ROC) analysis was performed to evaluate the predictive value of PRM_ADC_ for projecting patient 1-year survival post diagnosis.

## Results

### Semiautomated Workflow

An optimized workflow was developed for the PRM analysis of quantitative diffusion maps for glioma response assessment (Figure 1). First, a semiautomated segmentation algorithm allows for fast and objective delineation of tumor volumes at baseline and follow-up based on contrast-enhancing tumor (T1+C). Next, follow-up T1+C images were automatically coregistered to baseline T1+C. The resulting transformation was then applied to the calculated ADC maps. Individual voxels were then classified based on predetermined thresholds, generating relative volumes for each classification as a summary statistic, as well as a PRM map displayed as a color overlay on anatomical images.

Implementation of this workflow could be accomplished by locally installed software or cloud-based servers with access through a browser, and this workflow includes connectivity to PACS storage. This flexibility will allow users access to PRM algorithms without the need for purchasing expensive, high-performance computers. The output display (ie, PRM report) is designed to maximize the relevant information for the user in a succinct manner that is easy to read (Figure 2). In addition to the PRM_ADC_-relative volumes, the final generated report presents tumor-central slices with color PRM overlaid, the scatterplot that represents the distribution of ADC values from all voxels contained within the segmented tumor, and mean tumor ADC and tumor volume values from both time points. The output also includes a Digital Imaging and Communications in Medicine series of the full pretreatment T1+C volume with the color PRM results overlaid, downloaded directly to a PACS.

### Segmentation Evaluation

**Figure 4.**
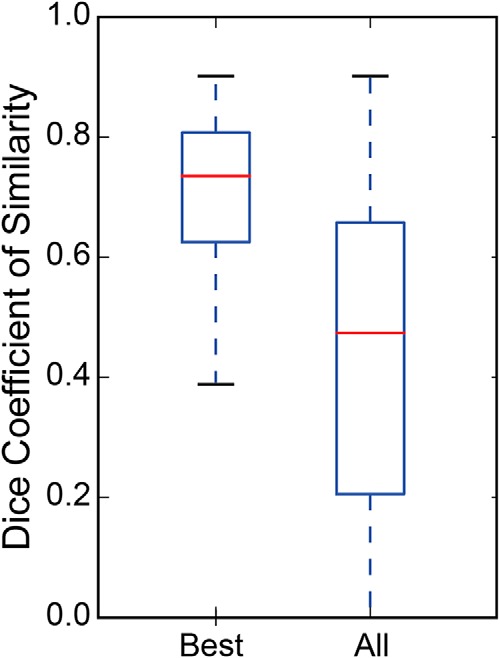
Dice coefficients comparing semiautomated tumor segmentations to radiologist-defined segmentations. Maximum Dice per patient resulted in good agreement between segmentations, left; however, use of all slice ROIs individually as seed regions resulted in poor agreement, right. This indicates that seed ROI positioning is critical for an acceptable segmentation. The seed ROI should be placed in the middle of a tumor, where the tumor area is the largest.

Manual delineation of the tumor volume by an experienced radiologist is time-intensive and subjective, often leading to interoperator variation (41). This has motivated the development of semiautomated and fully automated algorithms to provide fast and objective hands-off delineation of tumor volumes. Semiautomated segmentations used in the current PRM platform were consistently well placed, showing good agreement with manual segmentations (Figure 3). The location of a seed ROI (cyan line) for the illustrative example in Figure 3 was accomplished by a 2-dimensional dilation of the manual segmentation (blue line, top row) on slice 13, with the algorithm growing into a 3D VOI for use in quantitative analysis (green line, bottom row). The quality of the segmentation algorithm was found to be sensitive to positioning of the seed ROI (Figure 4). Here, the best agreement per patient between auto-segmentation and manual segmentations is high (mean DCS of 0.703 ± 0.151). These segmentations were found to result from seed ROIs generated in the middle of the tumor, where the area of the tumor was the largest. Comparisons including segmentations from all individual slice seeds for each patient resulted in a wide variation (DCS = 0.445 ± 0.256). Low DCS found in this analysis was typically found when seed ROIs were positioned at the end of the tumor volume, with a relatively low cross-sectional area. Seed positioning on larger cross sections of the tumor produced more stable results. We found an average difference in PRM_ADC+_ of 0.3% (±4.5%) between previous results and our semiautomated results.

**Figure 5.**
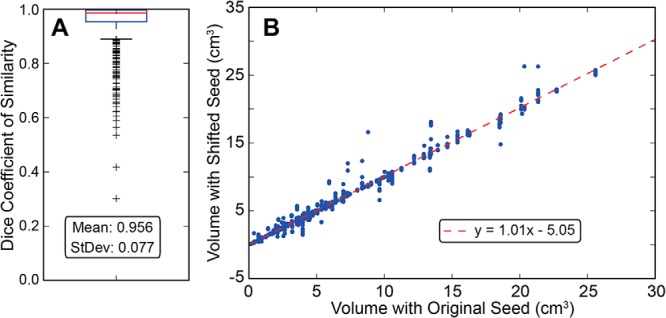
Robustness of semiautomated segmentation was evaluated using Dice coefficients between the best-slice seed segmentation and shifted-seed segmentations (A), showing little variation between segmentations. Resulting volumes were also found to remain consistent with varying segmentation seed (B).

Segmentation was further evaluated by applying a 1-voxel shift in each direction to the seed ROI that resulted in maximum DCS per patient. Resulting DCS and volumes were compared against original (maximum DCS) segmentations (Figure 5), showing good agreement between segmentations initiated with original versus shifted seeds (DCS = 0.96 ± 0.08; *R*_Vol_^2^ = 0.98).

### PRM Benchmarking

**Figure 6.**
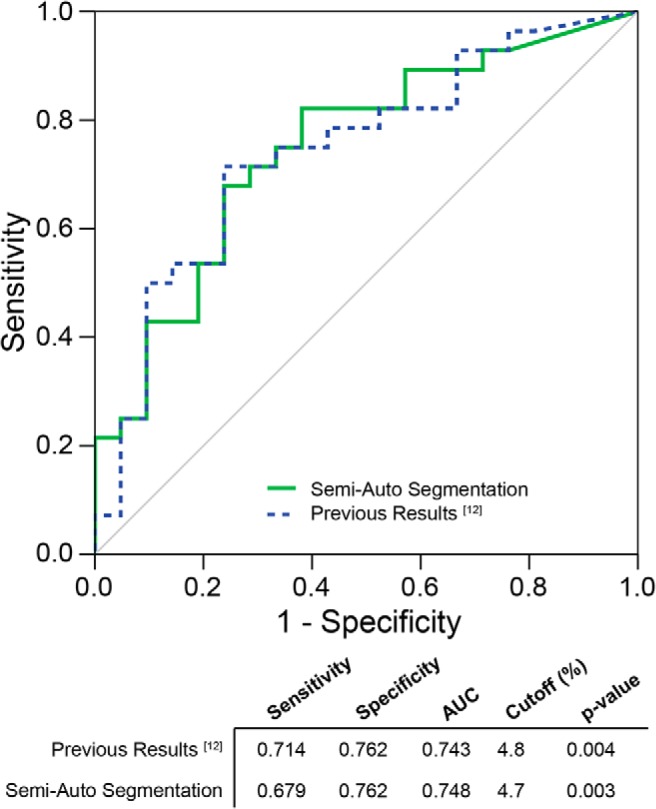
Receiver operator curve (ROC) analysis was used to compare final PRM_ADC+_ results against the same subjects' previous results (n = 49). Semiautomated segmentation was performed on previously coregistered images, followed by automated PRM classification. The ROC statistics are compared between the 2 in the table.

**Figure 7.**
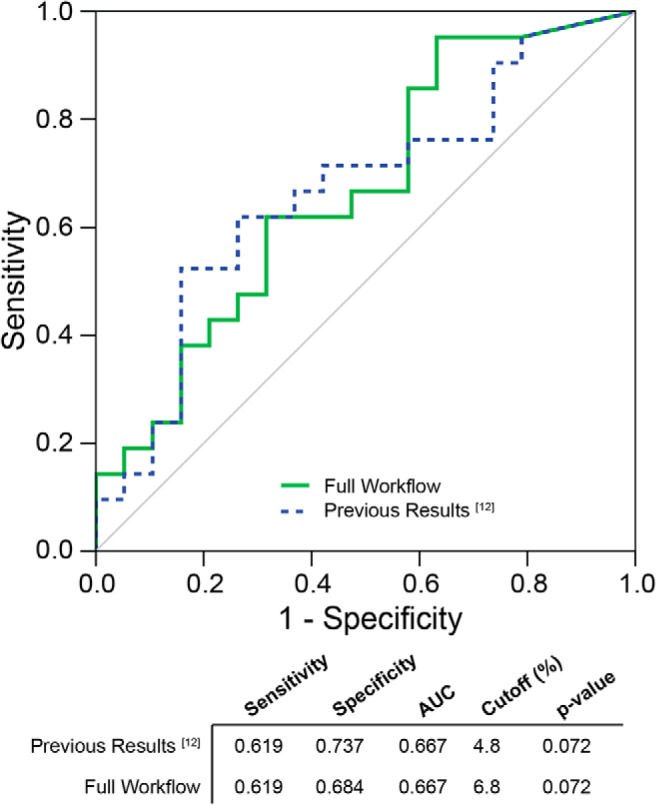
The ROC analysis was used to compare final PRM_ADC+_ results against the same subjects' previous results (n = 40). The full workflow was applied to available original imaging data, consisting of semiautomated segmentation, image coregistration, and PRM classification. ROC statistics are compared between the 2 in the table.

PRM results generated by the semiautomated software were benchmarked against previously published results (12). ROC analysis was performed in the previous study to find an optimal PRM_ADC+_ cutoff for prediction of 1-year survival. Subjects used in the ROC analysis were a subset of the original group. Semiautomated segmentation and classification using previously registered images (Figure 6) and the full semiautomated workflow results (Figure 7) both produced ROC characteristics that are comparable to the original analysis. Limited availability of the original imaging data for the full workflow analysis also limited the significance of the findings using that subset; however, our results closely matched those in the same subset of the original results.

## Discussion

Standard methods for determining GBM response to treatment currently rely on data obtained after 4 weeks following completion of the initial course of therapy. This approach potentially results in patients receiving weeks of ineffective therapy with associated systemic toxicities, delaying opportunities to improve patient outcomes through personalized, adaptive therapy. These limitations highlight the unmet clinical need for new, clinically translatable methods to assess response treatment efficacy early in the course of therapy. The use of PRM_ADC_ has been limited to research applications due in part to the absence of clinically streamlined software. The goal of the presented work was to develop a semiautomated PRM process for translation into standard clinical practice.

PRM_ADC_ requires 3D segmentation of the tumor volume, a cumbersome, time-consuming, and skill level-dependent process when done manually. Thus, automation of the segmentation process is critical for achieving consistent results in practical clinical implementation in the time-constrained environment of clinical radiology and patient care. After magnetic resonance images are acquired (DWI and T1+C), they can be automatically uploaded to the software platform, and the only user input necessary for this workflow is to designate the seed ROI for initialization of the segmentation algorithm. Using the presented semiautomated approach, only a rough contouring of the tumor boundaries on a single slice is required. We have shown good agreement between semiautomated and manual segmentations using the segmentation resulting from the best seed slice for each subject (DCS = 0.704). This maximum DCS generally resulted from a seed ROI placed on the slice with the largest cross-sectional tumor area (ie, on a tumor-central slice). For comparison, previous studies have determined the mean DCS between manually drawn segmentations of T1+C-enhancing glioma performed by separate readers to be 0.74 (42). The segmentation was also found to be quite robust, with minimal variation resulting from spatial shifts in the seed ROI (DCS = 0.96 ± 0.08). Further development of this workflow is ongoing, which will include a fully automated segmentation routine, but a robust method for this has not yet been implemented.

Workflow results were compared with previous results as a benchmark (12), first focusing on the segmentation, and then using the full workflow. The ROC results using preregistered images, semiautomated tumor segmentation, and automated PRM classification were consistent with previous results. Although previous analyses used images that were coregistered using an affine transformation, the presented workflow used a nonlinear warping transform to account for the nonrigid nature of these biological tissues. Although modest changes in tumor volume would be expected within the 3-week time frame of this analysis, the difference in coregistration has the potential to affect resulting PRM values and thus their clinical interpretation. Issues relating to image registration have been extensively discussed in the literature; however, they indicate that these deformable image-processing tools can be used effectively to coregister brain MRI data (10, 43). However, PRM_ADC+_ results were not found to differ greatly from previous results. This further confirmed that the semiautomated approach for tumor segmentation provides accurate results leading to minimal PRM differences. The ROC analysis was further used to evaluate the fully semiautomated workflow results (segmentation, registration, and PRM classification), again resulting in good agreement.

Overall, we have shown that the presented approach provides an easy-to-use solution for the clinical evaluation of DWI scans obtained from patients with glioma. The workflow requires minimal operator input (and thus minimal time) from the user and yields results consistent with previously published work. The final report generated for the user displays all information of interest from the analysis in a manner that is easily interpreted. Furthermore, the report also provides information to the user related to any encountered problems with the analysis, which would be apparent in the representative slice overlays and PRM scatterplot. Resulting segmentations and PRM overlays are also available for download in the Digital Imaging and Communications in Medicine standard format. Further development of the user interface and results will depend on direct user input once the software is implemented for routine clinical use.

In summary, the presented work demonstrates a clinically accessible PRM application for the assessment of glioma response to therapy. The streamlined process was shown to provide robust and comparable results to the time-intensive manual evaluation previously reported in the literature. Although evaluated only in the context of glioma response using DWI, this method remains flexible and broadly applicable with easy translation to other cancers and treatments (9, 12, 24–30), as well as other quantitative imaging modalities (35, 36, 43, 44). By providing a robust software platform, this work signifies a substantial advance toward translation of PRM imaging biomarkers into standard clinical practice and is a significant step toward allowing broad clinical adoption of PRM.
